# Supplementary figures and images for: Anaesthesia-Induced Transcriptomic Changes in the Context of Renal Ischemia Uncovered by the Use of a Novel Clamping Device
Source: Int J Mol Sci. 2021 Sep 11;22(18):9840. doi: 10.3390/ijms22189840 (PMC8464990; doi:10.3390/ijms22189840)

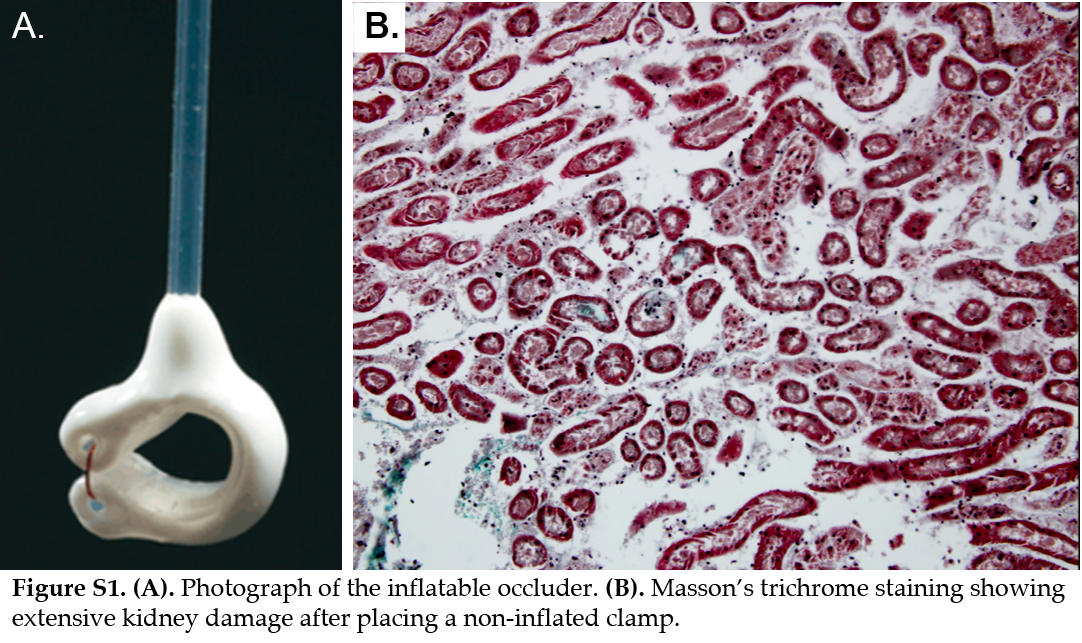

Supplement: Supplementary file 1 [file ijms-22-09840-s001.zip › Figure S1.tif]

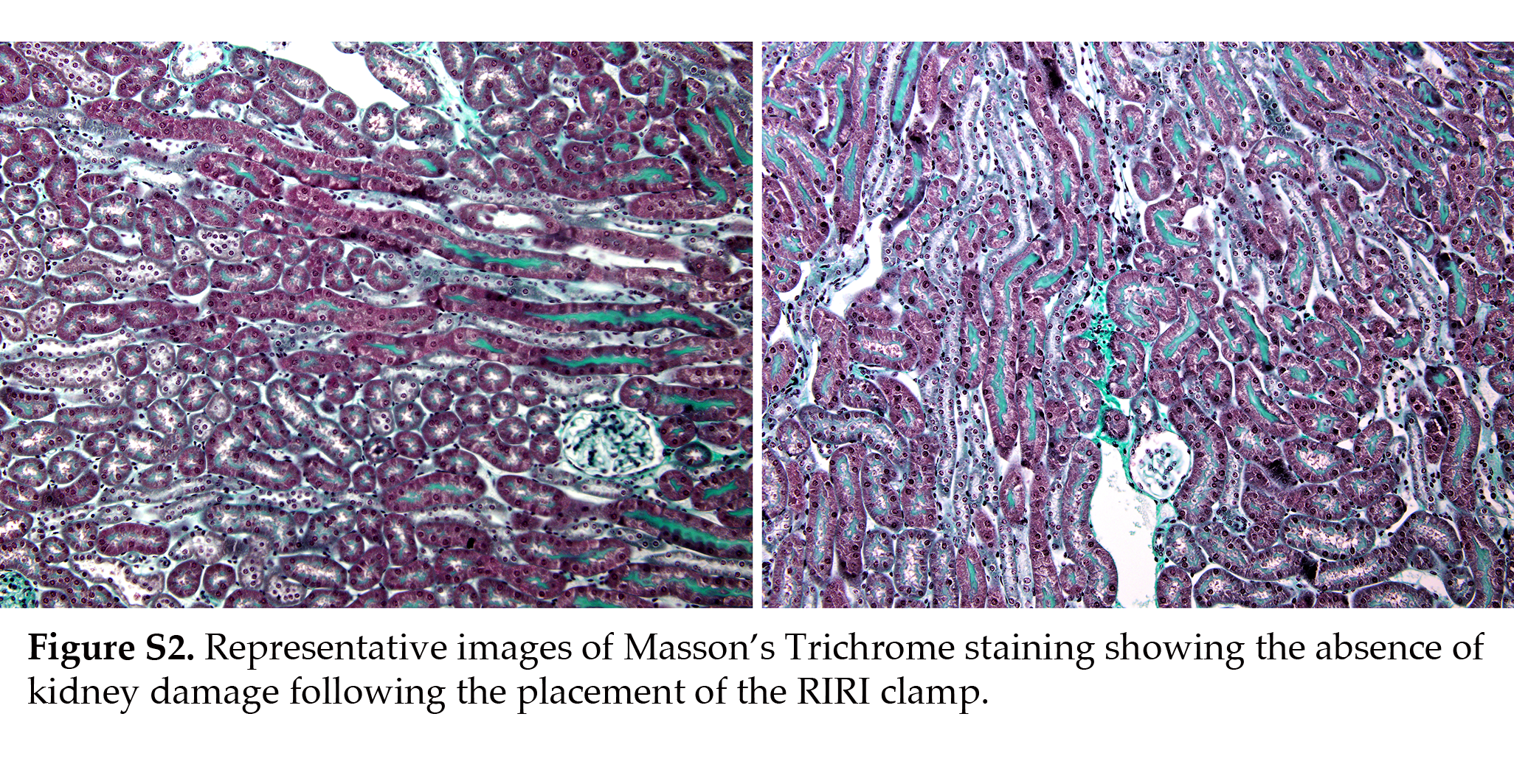

Supplement: Supplementary file 1 [file ijms-22-09840-s001.zip › Figure S2.tif]

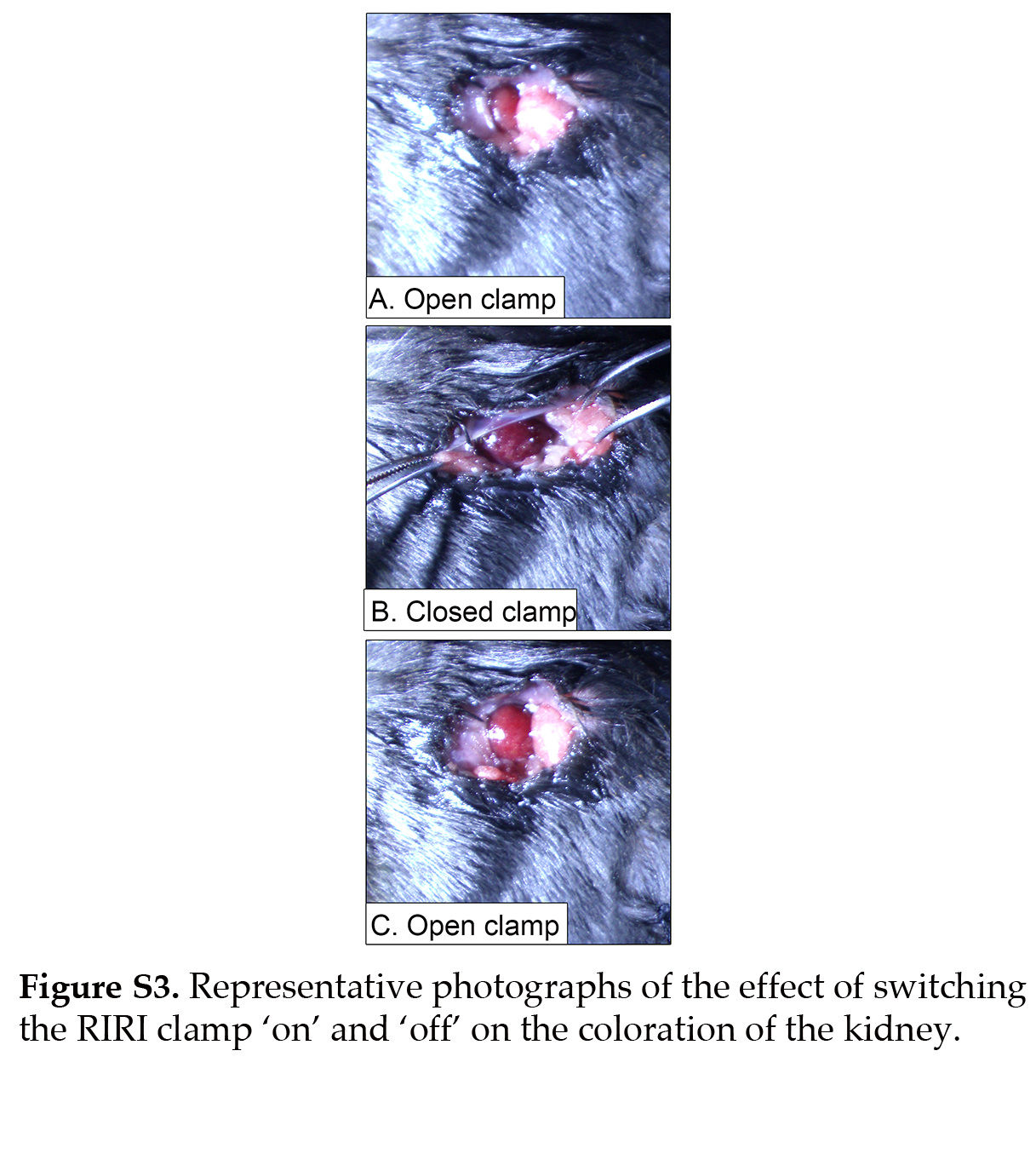

Supplement: Supplementary file 1 [file ijms-22-09840-s001.zip › Figure S3.tif]

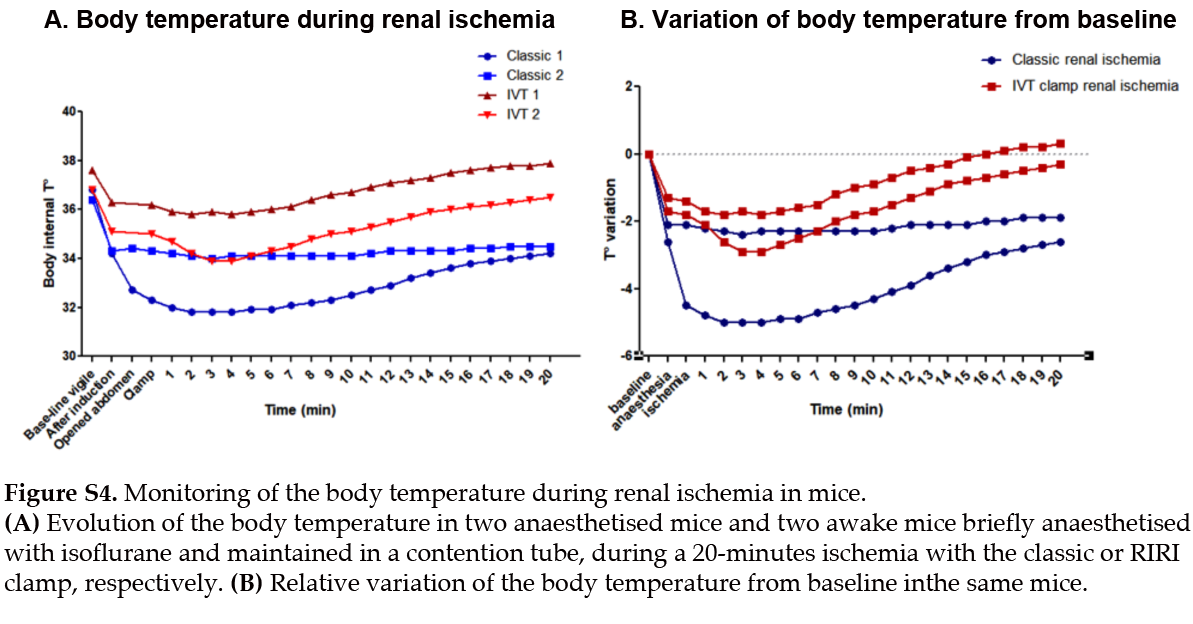

Supplement: Supplementary file 1 [file ijms-22-09840-s001.zip › Figure S4.tif]

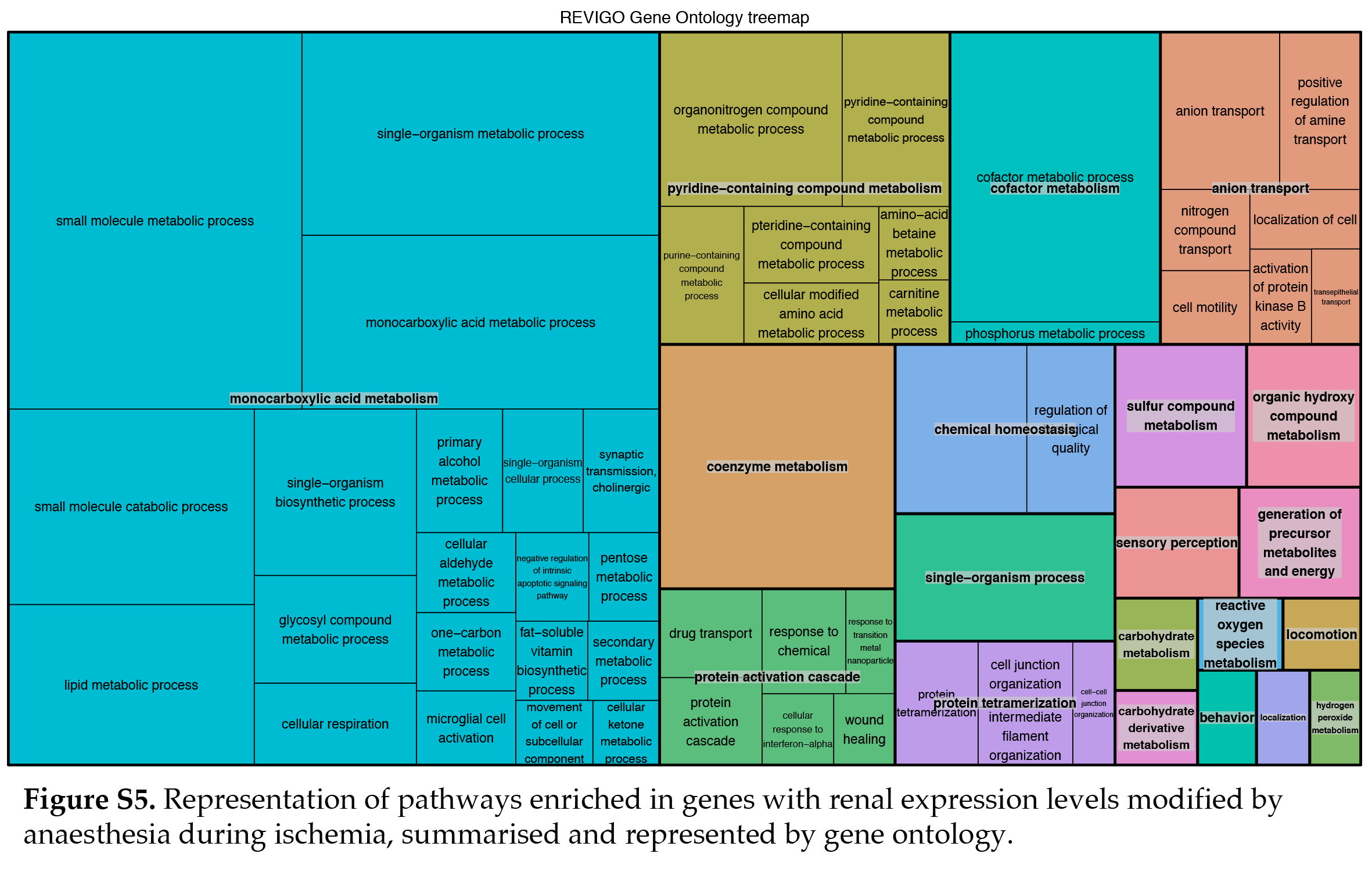

Supplement: Supplementary file 1 [file ijms-22-09840-s001.zip › Figure S5.tif]
