# Supplementary material for: Anaesthesia-Induced Transcriptomic Changes in the Context of Renal Ischemia Uncovered by the Use of a Novel Clamping Device
Source: Int J Mol Sci. 2021 Sep 11;22(18):9840. doi: 10.3390/ijms22189840 (PMC8464990; doi:10.3390/ijms22189840)
Supplement: Supplementary file 1 [file ijms-22-09840-s001.zip › Table S1.doc]

| **Primer Name** | **Primer Sequence** |
| --- | --- |
| HPRT-s  HPRT-as  GAPDH-s  GAPDH-as  GusB-s  GusB-as  Ubc-s  Ubc-as  RPL32-s  RPL32-as  KIM1-s  KIM1-as | cctcctcagaccgcttttt  aacctggttcatcatcgctaa  AGCTTGTCATCAACGGGAAG  TTTGATGTTAGTGGGGTCTCG  CTCTGGTGGCCTTACCTGAT  CAGTTGTTGTCACCTTCACCTC  AGCCCAGTGTTACCACCAAG  ACCCAAGAACAAGCACAAGG GCTGCCATCTGTTTTACGG  TGACTGGTGCCTGATGAACT  CCATGGTAGCCAAGGAGAGC  GCAAACCCAGAACGCAACTT |

**Table S1.** Sequence of the qPCR primers.
